# Crystal structure of *catena*-poly[[[tetra­aquacobalt(II)]-μ_2_-1,5-di­hydroxy­naphthalene-2,6-di­carboxyl­ato] di­methyl­formamide disolvate]

**DOI:** 10.1107/S2056989025000982

**Published:** 2025-02-07

**Authors:** Hitoshi Kumagai, Satoshi Kawata, Nobuhiro Ogihara

**Affiliations:** a Toyota Central R&D Labs., Inc., 41-1 Yokomichi, Nagakute, Aichi 480-1192, Japan; b Department of Chemistry Fukuoka University 8-19-1 Nanakuma Jonan-ku Fukuoka 814-0180 Japan; Tokyo University of Science, Japan

**Keywords:** cobalt, hydrogen bonding, C—H⋯π inter­action, dihy­droxy-naphthalenedi­carboxyl­ate, crystal structure

## Abstract

The asymmetric unit of the title compound, {[Co(C_12_H_6_O_6_)(H_2_O)_4_]·2C_3_H_7_NO}_*n*_ or {[Co(H_2_dondc)(H_2_O)_4_]·2DMF}_*n*_, comprises half of a Co^II^ ion, half of a 1,5-di­hydroxy­naphthalene-2,6-di­carboxyl­ate dianion (H_2_dondc^2−^), two water mol­ecules and a di­methyl­formamide (DMF) mol­ecule. The Co^II^ ion, which is located on a crystallographic inversion center, exhibits a distorted six-coord­inated octa­hedral geometry with two oxygen atoms of the H_2_dondc^2−^ ligand and four oxygen atoms of the water mol­ecules. The carboxyl­ate group is almost coplanar with the naphthalene moiety and shows monodentate coordination to the Co^II^ ion. The Co^II^ ions are bridged by the H_2_dondc^2−^ ligand to form a one-dimensional chain. The hy­droxy groups of the ligand have intra-chain hydrogen bonding inter­actions with coordinated water mol­ecules. The coordinated water mol­ecules exhibit not only intra-chain hydrogen bonding inter­actions, but also inter-chain hydrogen-bonding inter­actions. The chains are connected by inter-chain hydrogen-bonding inter­actions and are arranged in parallel to form a two-dimensional network. The chains are further connected by inter-chain hydrogen-bonding inter­actions *via* the DMF mol­ecules and C—H⋯π inter­actions to give a three-dimensional network.

## Abstract 2

The asymmetric unit of the title compound, {[Co(C_12_H_6_O_6_)(H_2_O)_4_]·2C_3_H_7_NO}_*n*_ or {[Co(H_2_dondc)(H_2_O)_4_]·2DMF}_*n*_, comprises half of a Co^II^ ion, half of a 1,5-di­hydroxy­naphthalene-2,6-di­carboxyl­ate dianion (H_2_dondc^2−^), two water mol­ecules and a di­methyl­formamide (DMF) mol­ecule. The key feature of the structure is a three-dimensional hydrogen-bonding network that consists of one-dimensional (1D) coordination chains built up by CoO_6_ octa­hedra bridged by H_2_dondc^2−^ ligands and inter­chain O–H⋯O hydrogen-bonding inter­actions.

## 1. Chemical context

Metal–organic frameworks (MOFs) or coordination polymers (CPs) are being actively investigated due to their applications in gas adsorption, separation and catalysis (Cheetham *et al.*, 1999 ▸; Eddaoudi *et al.*, 2002 ▸; Kitagawa *et al.*, 2004 ▸). Polycarboxyl­ate ligands such as benzene­dicarboxyl­ate (bdc^2−^ dianion), also known as a terephthalate dianion, are well-known linkers that yield functional materials (Furukawa *et al.*, 2010 ▸; Kurmoo, 2009 ▸). We have not only prepared electrode materials using the terephthalate dianion and its derivatives (Ogihara *et al.*, 2014 ▸, 2021 ▸,2023 ▸; Yasuda & Ogihara, 2014 ▸), but also magnetic materials that involve polycarboxyl­ates in which the number of carboxyl­ate groups and the distances between carboxyl­ate groups vary systematically (Kumagai *et al.*, 2001 ▸, 2002 ▸; Kurmoo *et al.*, 2001 ▸, 2003 ▸). The functionalization of an organic ligand provides further coordination capabilities, reaction centers, and inter­action sites for specific functions. The 2,5-dihy­droxy-1,4-benzene­dicarb­oxy­lic acid (2,5-H_4_dobdc) ligand is a 1,4-benzene­dicarb­oxy­lic acid derivative with two hy­droxy groups introduced as functional groups and functional MOFs with this ligand have been reported (Caskey *et al.*, 2008 ▸; Cozzolino *et al.*, 2014 ▸; Geier *et al.*, 2013 ▸; Maurice *et al.*, 2013 ▸; Queen *et al.*, 2014 ▸). 1,5-Di­hydroxy­naphthalene-2,6-di­carboxyl­ate (H_4_dondc) is an analogue of 2,5-H_4_dobdc. The H_4_dondc ligand can be deprotonated to give four available charges (1- to 4-); however, only metal complexes of the 4- anion have been reported (Dietzel *et al.*, 2020 ▸; Yeon *et al.*, 2015 ▸). In this contribution, we have focused on the use of 1,5-di­hydroxy­naphthalene-2,6-di­carboxyl­ate (H_2_dondc^2−^) in the synthesis of a Co^II^–H_2_dondc^2−^ dianion system and report on the single-crystal structure of [Co(H_2_dondc)(H_2_O)_4_]·2DMF in which the ligand is a 2- anion. This is a new structure of the metal complex synthesized from H_4_dondc.
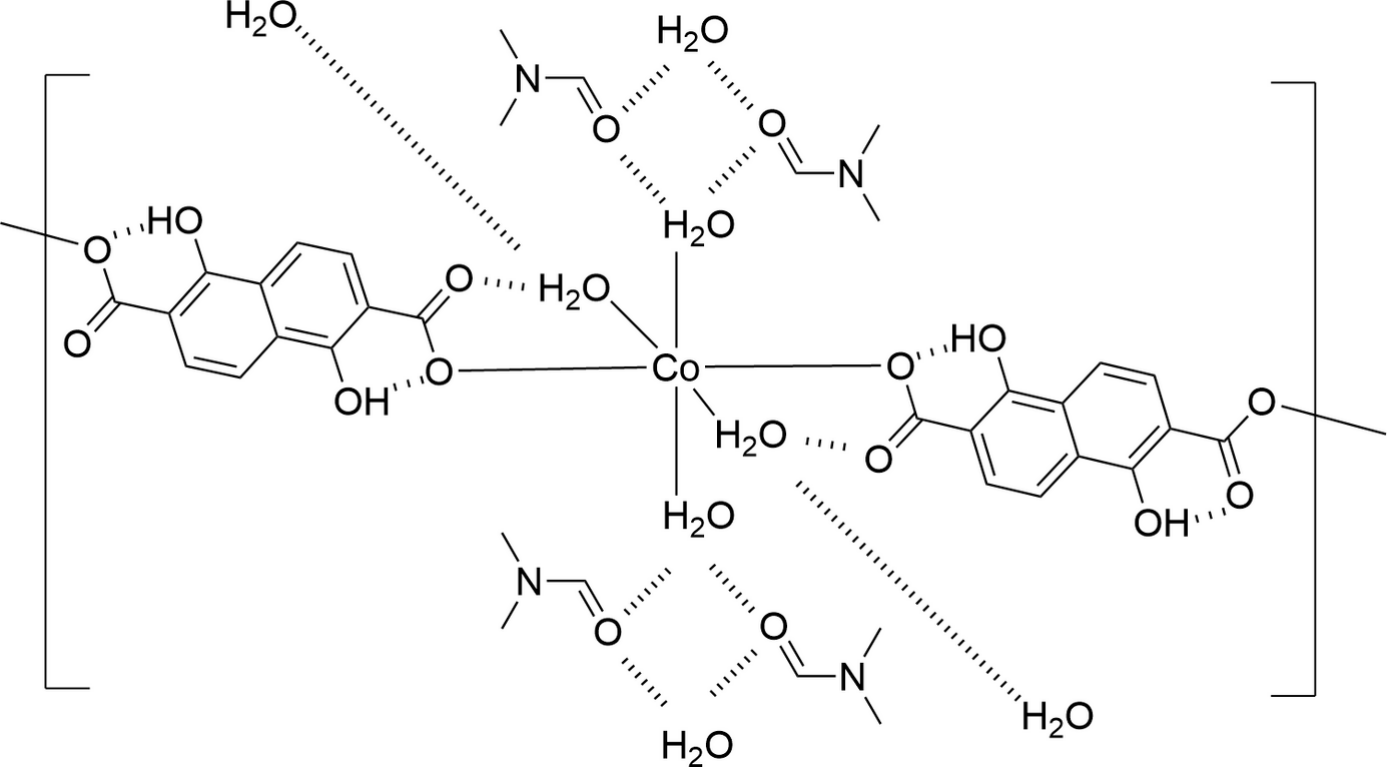


## 2. Structural commentary

**Figure 1:**
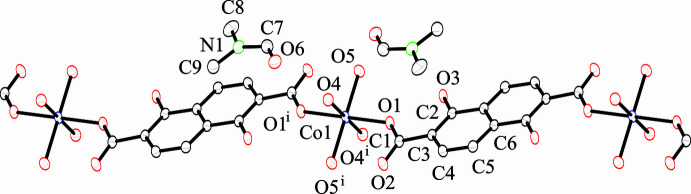
One-dimensional chain structure of the title compound with the atom-labeling scheme and 50% probability displacement ellipsoids. Hydrogen atoms are omitted for clarity. [Symmetry code: (i) −*x* + 1, −*y* + 1, −*z* + 1.]

The title compound, [Co(H_2_dondc)(H_2_O)_4_]·2DMF, consists of a Co^II^ ion, a 1,5-di­hydroxy­naphthalene-2,6-di­carboxyl­ate dianion (H_2_dondc^2−^), four water mol­ecules and two DMF mol­ecules. The Co^II^ ion lies on a crystallographic inversion center and its asymmetric unit consists of half of a Co^II^ ion, half of a H_2_dondc^2−^ ligand, two water mol­ecules and a DMF mol­ecule. The key feature of the structure is a three-dimensional (3D) hydrogen-bonding network that consists of one-dimensional (1D) coordination chains built up by CoO_6_ octa­hedra bridged by H_2_dondc^2−^ ligands and inter­chain O–H⋯O hydrogen-bonding inter­actions. Fig. 1 ▸ shows the 1D chain structure of [Co(H_2_dondc)(H_2_O)_4_] and DMF mol­ecules with the numbering scheme. The Co^II^ ion occupies a crystallographic inversion center; therefore, each pair of H_2_dondc^2−^ ligands and water mol­ecules coordinate *trans* to each other to form a linear chain. The Co—O1 (carboxyl­ate) bond length [2.0750 (9) Å] in the title compound is shorter than the Co—O (H_2_O) bond lengths [2.0931 (10) and 2.1023 (10) Å], which is indicative of the compressed octa­hedral geometry of the Co^II^ ion. The Co⋯Co separation defined by Co–H_2_dondc^2−^–Co connectivity within the chain is 13.27 Å. We have reported CPs that consist of tetra­halogenated terephthalate dianions as bridging ligands in which the carboxyl­ate groups exhibited monodentate coordination similar to the title compound. The Co⋯Co separation distance (*ca*. 11 Å) is shorter than that observed in the title compound (Kumagai *et al.*, 2021 ▸), which is due to the long naphthalene backbone. The carboxyl­ate group exhibits monodentate coordination, and the dihedral angle between the carboxyl­ate group and the naphthalene ring system is slightly tilted with an O1—C1—C3—C4 torsion angle of 171.94 (11)°. Non-coordinated oxygen atoms of the carboxyl­ate groups show intra-chain hydrogen-bonding inter­actions with coordinated water mol­ecules. The non-coordinated oxygen atoms of the carboxyl­ate groups act as hydrogen-bond acceptors and coordinated water mol­ecules act as hydrogen-bond donors. The H_2_dondc^2−^ ligand binds to the Co^II^ ion solely through its carboxyl­ate oxygen atoms and the phenolic hydroxyl groups show no coordination bonding to Co^II^ ions. The hydroxyl groups act as hydrogen-bond donors and undergo hydrogen bonding with the coordinated carboxyl­ate oxygen atoms as hydrogen-bond acceptors. The H_4_dondc ligand can be deprotonated to give four available charges (1− to 4−), although only metal complexes of the 4− anion have been reported so far. Both the hy­droxy and carb­oxy­lic acid groups of the ligand are deprotonated to give a 4− anion and the resultant oxido and carboxyl­ate groups are coordinated by metal ions to give the *M*_2_(dondc) composition (*M* = Mn, Mg, Ni, Co) and a honeycomb-like structure (Dietzel *et al.*, 2020 ▸; Yeon *et al.*, 2015 ▸).

## 3. Supra­molecular features

**Figure 2:**
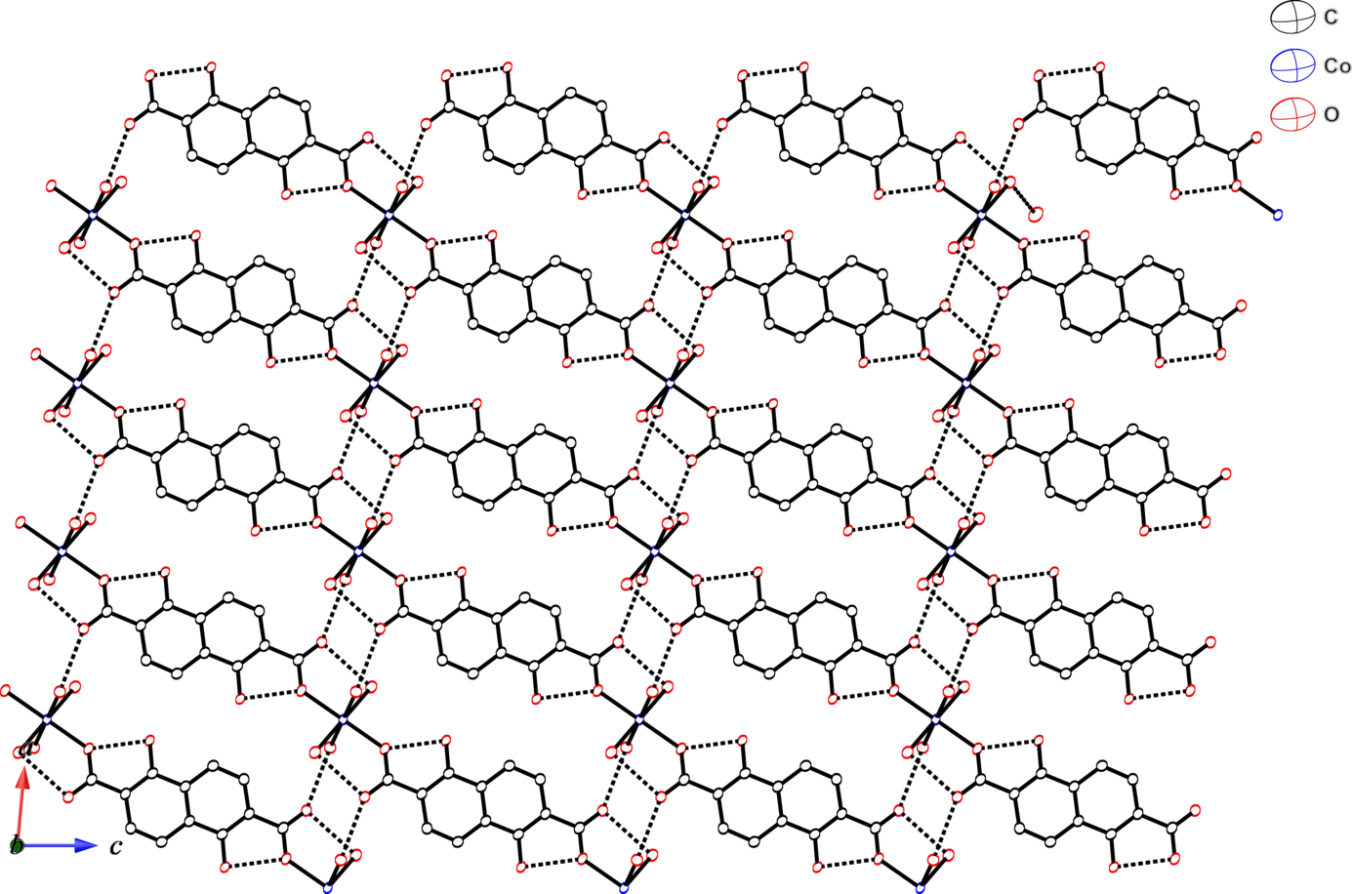
View of the two-dimensional hydrogen-bonding network with inter- and intra-chain hydrogen-bonding inter­actions. Dashed lines represent hydrogen bonds.

**Figure 3:**
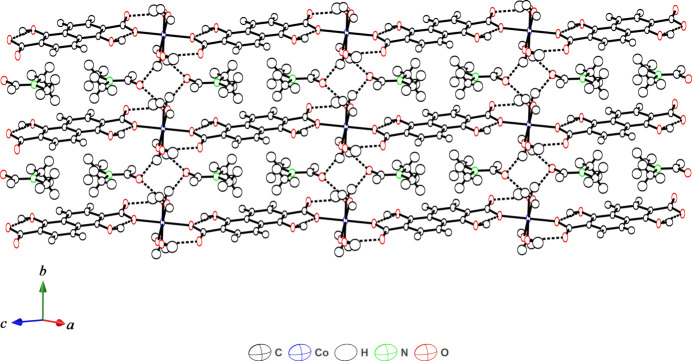
Inter-chain hydrogen-bonding inter­actions *via* DMF mol­ecules. Dashed lines represent inter-chain hydrogen bonds.

The coordinated water mol­ecules in the crystal structure act as hydrogen-bonding donors and the oxygen atoms of the DMF mol­ecules act as hydrogen-bonding acceptors (Table 1 ▸). The coordinated water mol­ecule (O4) shows two types of hydrogen-bonding inter­actions. One is an inter-chain hydrogen-bonding inter­action between the non-coordinated oxygen atom of the adjacent chain to give a two-dimensional hydrogen-bonding network in which the chains are arranged in parallel when viewed along the *b*-axis direction (Fig. 2 ▸). The other is an inter-mol­ecular hydrogen-bonding inter­action between the DMF and water mol­ecules. The coordinated water mol­ecule (O5) exhibits not only inter-chain hydrogen-bonding inter­actions with the oxygen atom of a DMF mol­ecule but also intra-chain hydrogen-bonding inter­actions with an oxygen atom (O2) of the carboxyl­ate group not bound to the Co^II^ ions. The DMF mol­ecule acts as a hydrogen-bond acceptor for both O4 and O5 in different chains to yield a hydrogen-bonding network (Fig. 3 ▸). The C3⋯C8 distance of 3.497 (2) Å between the carbon atoms of DMF mol­ecules and the naphthalene ring system, and the C8⋯centroid distance of 3.53 Å are indicative of some degree of C—H⋯π inter­action (Nishio, 2011 ▸; Nishio *et al.*, 2009 ▸). Therefore, the DMF mol­ecules are held in between the one-dimensional chains by hydrogen-bonding inter­actions and C—H⋯π inter­actions(Fig. S1 in the supporting information). The presence of DMF mol­ecules between the chains prevents π–π stacking inter­actions between the planar naphthalene moieties and the two naphthalene moieties are 6.96 Å apart.

## 4. Database survey

Although a search of the Sci Finder database for structures with a H_2_dondc^2−^ and Co^II^ ion resulted in no complete matches, partially matched structures were found. They are metal complexes composed of a Mn^II^ ion and a dondc^4−^ ligand that form a three-dimensional network consisting of hexa­gonal channels (CADYOZ and CADYUF; Dietzel *et al.*, 2020 ▸). A search of the Web of Science database for the keywords 2,6-naphthalenedi­carb­oxy­lic acid and 1,5-dihy­droxy- led to lanthanide-based compounds [DUDXOS (La) and DUDXUY (Ce); Mahmoud *et al.*, 2020 ▸] and an Mg^II^ compound (Yeon *et al.*, 2015 ▸). The structure of the Mg^II^ compound is similar to that of the Mn^II^ compound.

## 5. Synthesis and crystallization

Cobalt(II) nitrate hexa­hydrate (0.12 g, 0.4 mmol) and H_4_dondc were dissolved in an ethanol (10 mL)–*N*,*N*-di­methyl­formamide (20 mL) mixture. The mixture was placed in the Teflon liner of an autoclave, sealed, and heated at 353 K for two days. The mixture was then cooled to room temperature. Pink crystals were obtained and one of these crystals was used for single-crystal X-ray crystallography analysis.

## 6. Refinement

Crystal data, data collection and structure refinement details are summarized in Table 2 ▸. The non-hydrogen atoms were refined anisotropically. The hydrogen atoms attached to oxygen atoms of the ligand and water mol­ecules were extracted from difference-Fourier maps. Other hydrogen atoms were placed in idealized positions (C—H = 0.95–0.98 Å) and refined using a riding model with *U*_iso_(H) = 1.2*U*_eq_(C).

## Supplementary Material

Crystal structure: contains datablock(s) I. DOI: 10.1107/S2056989025000982/jp2016sup1.cif

Structure factors: contains datablock(s) I. DOI: 10.1107/S2056989025000982/jp2016Isup2.hkl

Supporting information file. DOI: 10.1107/S2056989025000982/jp2016Isup3.cdx

Supporting FigS1. View of the CH-pi interactions. Dashed lines represent CH-pi interactions. DOI: 10.1107/S2056989025000982/jp2016sup4.tif

Supporting FigS2. View of the CH-pi interactions along b-axis. DOI: 10.1107/S2056989025000982/jp2016sup5.tif

Supporting FigS3. View of the CH-pi interactions along a-axis. Dashed lines represent CH-pi interactions. DOI: 10.1107/S2056989025000982/jp2016sup6.tif

CCDC reference: 2421049

Additional supporting information:  crystallographic information; 3D view; checkCIF report

## Figures and Tables

**Table 1:** Hydrogen-bond geometry (Å, °)

*D*—H⋯*A*	*D*—H	H⋯*A*	*D*⋯*A*	*D*—H⋯*A*
O3—H3⋯O1	0.78 (2)	1.81 (2)	2.5168 (13)	149.1 (19)
O4—H1⋯O6	0.76 (2)	1.99 (2)	2.7449 (16)	178 (2)
O4—H2⋯O2^i^	0.78 (2)	1.96 (2)	2.7284 (15)	168 (2)
O5—H6⋯O2^ii^	0.79 (3)	2.00 (3)	2.7182 (14)	151 (2)
O5—H10⋯O6^iii^	0.85 (2)	1.95 (2)	2.7836 (15)	166 (2)
C8—H8*C*⋯C3^ii^	0.98	2.85	3.497 (2)	124

Symmetry codes: (i) 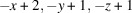; (ii) 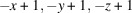; (iii) 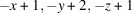.

**Table 2:** Experimental details

Crystal data	
Chemical formula	[Co(C_12_H_6_O_6_)(H_2_O)_4_]·2C_3_H_7_NO
*M*_r_	523.35
Crystal system, space group	Triclinic, *P* 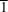
Temperature (K)	110
*a*, *b*, *c* (Å)	6.886 (1), 6.945 (1), 12.0366 (15)
α, β, γ (°)	85.543 (5), 84.371 (5), 83.981 (5)
*V* (Å^3^)	568.37 (14)
*Z*	1
Radiation type	Mo *K*α
μ (mm^−1^)	0.82
Crystal size (mm)	0.25 × 0.25 × 0.10

Data collection	
Diffractometer	Rigaku R-AXIS RAPID
Absorption correction	Multi-scan (*ABSCOR*; Rigaku, 1995 ▸)
*T*_min_, *T*_max_	0.760, 0.922
No. of measured, independent and observed [*I* > 2σ(*I*)] reflections	9228, 2595, 2413
*R*_int_	0.027
(sin θ/λ)_max_ (Å^−1^)	0.649

Refinement	
*R*[*F*^2^ > 2σ(*F*^2^)], *wR*(*F*^2^), *S*	0.028, 0.074, 1.11
No. of reflections	2595
No. of parameters	173
H-atom treatment	H atoms treated by a mixture of independent and constrained refinement
Δρ_max_, Δρ_min_ (e Å^−3^)	0.62, −0.24

Computer programs: *RAPID-AUTO* (Rigaku, 2002 ▸), *SHELXT2014/5* (Sheldrick, 2015*a* ▸), *SHELXL2018/3* (Sheldrick, 2015*b* ▸) and *Yadokari-XG* (Wakita, 2001 ▸).

## supplementary crystallographic information

### catena-Poly[[[tetraaquacobalt(II)]-µ2-1,5-dihydroxynaphthalene-2,6-dicarboxylato] dimethylformamide disolvate] Crystal data

**Table:**

[Co(C_12_H_6_O_6_)(H_2_O)_4_]·2C_3_H_7_NO	*Z* = 1
*M**_r_* = 523.35	*F*(000) = 273
Triclinic, *P*1	*D*_x_ = 1.529 Mg m^−^^3^
*a* = 6.886 (1) Å	Mo *K*α radiation, λ = 0.71075 Å
*b* = 6.945 (1) Å	Cell parameters from 7544 reflections
*c* = 12.0366 (15) Å	θ = 3.3–27.5°
α = 85.543 (5)°	µ = 0.82 mm^−^^1^
β = 84.371 (5)°	*T* = 110 K
γ = 83.981 (5)°	Block, orange
*V* = 568.37 (14) Å^3^	0.25 × 0.25 × 0.10 mm

### catena-Poly[[[tetraaquacobalt(II)]-µ2-1,5-dihydroxynaphthalene-2,6-dicarboxylato] dimethylformamide disolvate] Data collection

**Table:**

Rigaku R-AXIS RAPID diffractometer	2413 reflections with *I* > 2σ(*I*)
ω scans	*R*_int_ = 0.027
Absorption correction: multi-scan (ABSCOR; Rigaku, 1995)	θ_max_ = 27.5°, θ_min_ = 3.3°
*T*_min_ = 0.760, *T*_max_ = 0.922	*h* = −8→8
9228 measured reflections	*k* = −9→9
2595 independent reflections	*l* = −15→14

### catena-Poly[[[tetraaquacobalt(II)]-µ2-1,5-dihydroxynaphthalene-2,6-dicarboxylato] dimethylformamide disolvate] Refinement

**Table:**

Refinement on *F*^2^	Primary atom site location: structure-invariant direct methods
Least-squares matrix: full	Secondary atom site location: difference Fourier map
*R*[*F*^2^ > 2σ(*F*^2^)] = 0.028	Hydrogen site location: inferred from neighbouring sites
*wR*(*F*^2^) = 0.074	H atoms treated by a mixture of independent and constrained refinement
*S* = 1.11	*w* = 1/[σ^2^(*F*_o_^2^) + (0.0456*P*)^2^ + 0.1029*P*] where *P* = (*F*_o_^2^ + 2*F*_c_^2^)/3
2595 reflections	(Δ/σ)_max_ < 0.001
173 parameters	Δρ_max_ = 0.62 e Å^−^^3^
0 restraints	Δρ_min_ = −0.24 e Å^−^^3^

### catena-Poly[[[tetraaquacobalt(II)]-µ2-1,5-dihydroxynaphthalene-2,6-dicarboxylato] dimethylformamide disolvate] Special details

**Table:**

Geometry. All esds (except the esd in the dihedral angle between two l.s. planes) are estimated using the full covariance matrix. The cell esds are taken into account individually in the estimation of esds in distances, angles and torsion angles; correlations between esds in cell parameters are only used when they are defined by crystal symmetry. An approximate (isotropic) treatment of cell esds is used for estimating esds involving l.s. planes.

### catena-Poly[[[tetraaquacobalt(II)]-µ2-1,5-dihydroxynaphthalene-2,6-dicarboxylato] dimethylformamide disolvate] Fractional atomic coordinates and isotropic or equivalent isotropic displacement parameters (Å^2^)

**Table:**

	*x*	*y*	*z*	*U*_iso_*/*U*_eq_	
Co1	0.500000	0.500000	0.500000	0.01471 (10)	
O1	0.67306 (14)	0.46799 (15)	0.35020 (7)	0.0190 (2)	
O2	0.96008 (13)	0.33348 (15)	0.40363 (8)	0.0210 (2)	
O3	0.62385 (13)	0.54304 (15)	0.14635 (8)	0.0179 (2)	
H3	0.597 (3)	0.535 (3)	0.2110 (17)	0.030 (5)*	
O4	0.66579 (15)	0.72005 (16)	0.53529 (8)	0.0191 (2)	
O5	0.30573 (15)	0.69999 (16)	0.41516 (8)	0.0199 (2)	
O6	0.50238 (16)	0.98724 (16)	0.68258 (9)	0.0271 (2)	
N1	0.22328 (19)	1.01767 (18)	0.80010 (10)	0.0243 (3)	
C1	0.85613 (19)	0.41209 (19)	0.33048 (10)	0.0156 (3)	
C2	0.81861 (18)	0.50097 (18)	0.12707 (10)	0.0142 (2)	
C3	0.94074 (18)	0.44228 (18)	0.21177 (10)	0.0141 (2)	
C4	1.14541 (18)	0.40885 (19)	0.18353 (10)	0.0161 (3)	
H4	1.229079	0.371976	0.241383	0.019*	
C5	1.22599 (18)	0.42820 (19)	0.07517 (11)	0.0160 (3)	
H5	1.363922	0.406279	0.058549	0.019*	
C6	1.10219 (17)	0.48131 (18)	−0.01250 (10)	0.0138 (2)	
C7	0.3219 (2)	1.0217 (2)	0.70011 (12)	0.0250 (3)	
H7	0.249193	1.053293	0.636960	0.030*	
C8	0.0115 (2)	1.0587 (2)	0.81476 (16)	0.0349 (4)	
H8A	−0.039095	1.089840	0.741516	0.042*	
H8B	−0.022584	1.169262	0.861194	0.042*	
H8C	−0.046646	0.944645	0.851466	0.042*	
C9	0.3257 (3)	0.9726 (2)	0.90075 (13)	0.0345 (4)	
H9A	0.299710	1.081591	0.948870	0.041*	
H9B	0.466950	0.950019	0.879845	0.041*	
H9C	0.279186	0.855710	0.941191	0.041*	
H1	0.619 (3)	0.795 (3)	0.5747 (18)	0.034 (6)*	
H2	0.769 (3)	0.690 (3)	0.5561 (16)	0.028 (5)*	
H6	0.214 (4)	0.728 (4)	0.458 (2)	0.057 (7)*	
H10	0.351 (3)	0.806 (3)	0.3928 (17)	0.037 (5)*	

### catena-Poly[[[tetraaquacobalt(II)]-µ2-1,5-dihydroxynaphthalene-2,6-dicarboxylato] dimethylformamide disolvate] Atomic displacement parameters (Å^2^)

**Table:**

	*U*^11^	*U*^22^	*U*^33^	*U*^12^	*U*^13^	*U*^23^
Co1	0.01037 (14)	0.02391 (15)	0.00905 (13)	−0.00095 (9)	0.00171 (8)	−0.00044 (9)
O1	0.0131 (4)	0.0315 (5)	0.0112 (4)	0.0002 (4)	0.0027 (3)	−0.0011 (4)
O2	0.0125 (4)	0.0359 (6)	0.0131 (4)	−0.0005 (4)	0.0008 (3)	0.0026 (4)
O3	0.0106 (4)	0.0320 (5)	0.0102 (4)	−0.0008 (4)	0.0025 (3)	−0.0015 (4)
O4	0.0128 (5)	0.0269 (5)	0.0175 (5)	−0.0001 (4)	−0.0010 (4)	−0.0032 (4)
O5	0.0142 (5)	0.0277 (5)	0.0161 (5)	−0.0004 (4)	0.0018 (4)	0.0024 (4)
O6	0.0247 (5)	0.0275 (5)	0.0273 (6)	−0.0020 (4)	0.0060 (4)	−0.0019 (4)
N1	0.0268 (7)	0.0218 (6)	0.0231 (6)	−0.0044 (5)	0.0058 (5)	−0.0024 (5)
C1	0.0133 (6)	0.0206 (6)	0.0131 (6)	−0.0046 (5)	0.0018 (4)	−0.0028 (5)
C2	0.0112 (6)	0.0176 (6)	0.0139 (6)	−0.0034 (4)	0.0025 (4)	−0.0036 (5)
C3	0.0129 (6)	0.0172 (6)	0.0121 (6)	−0.0023 (5)	0.0023 (4)	−0.0027 (4)
C4	0.0133 (6)	0.0215 (6)	0.0134 (6)	−0.0024 (5)	−0.0008 (4)	−0.0009 (5)
C5	0.0110 (6)	0.0217 (6)	0.0152 (6)	−0.0017 (5)	0.0009 (4)	−0.0027 (5)
C6	0.0122 (6)	0.0165 (6)	0.0128 (6)	−0.0028 (5)	0.0016 (4)	−0.0029 (5)
C7	0.0272 (8)	0.0252 (7)	0.0222 (7)	−0.0049 (6)	0.0021 (6)	−0.0009 (6)
C8	0.0272 (8)	0.0304 (8)	0.0463 (10)	−0.0085 (7)	0.0131 (7)	−0.0098 (7)
C9	0.0504 (11)	0.0291 (8)	0.0220 (8)	0.0001 (7)	0.0024 (7)	−0.0014 (6)

### catena-Poly[[[tetraaquacobalt(II)]-µ2-1,5-dihydroxynaphthalene-2,6-dicarboxylato] dimethylformamide disolvate] Geometric parameters (Å, º)

**Table:**

Co1—O1^i^	2.0750 (9)	N1—C9	1.458 (2)
Co1—O1	2.0750 (9)	C1—C3	1.4969 (17)
Co1—O4^i^	2.0931 (10)	C2—C3	1.3945 (18)
Co1—O4	2.0931 (10)	C2—C6^ii^	1.4334 (17)
Co1—O5	2.1023 (10)	C3—C4	1.4161 (17)
Co1—O5^i^	2.1023 (10)	C4—C5	1.3687 (17)
O1—C1	1.2842 (16)	C4—H4	0.9500
O2—C1	1.2460 (17)	C5—C6	1.4235 (18)
O3—C2	1.3436 (15)	C5—H5	0.9500
O3—H3	0.78 (2)	C6—C6^ii^	1.411 (2)
O4—H1	0.76 (2)	C7—H7	0.9500
O4—H2	0.78 (2)	C8—H8A	0.9800
O5—H6	0.79 (3)	C8—H8B	0.9800
O5—H10	0.85 (2)	C8—H8C	0.9800
O6—C7	1.2409 (19)	C9—H9A	0.9800
N1—C7	1.3226 (19)	C9—H9B	0.9800
N1—C8	1.452 (2)	C9—H9C	0.9800
			
O1^i^—Co1—O1	180.0	O3—C2—C6^ii^	116.24 (11)
O1^i^—Co1—O4^i^	89.40 (4)	C3—C2—C6^ii^	120.54 (11)
O1—Co1—O4^i^	90.60 (4)	C2—C3—C4	119.02 (11)
O1^i^—Co1—O4	90.60 (4)	C2—C3—C1	120.40 (11)
O1—Co1—O4	89.40 (4)	C4—C3—C1	120.57 (11)
O4^i^—Co1—O4	180.0	C5—C4—C3	121.81 (12)
O1^i^—Co1—O5	91.09 (4)	C5—C4—H4	119.1
O1—Co1—O5	88.91 (4)	C3—C4—H4	119.1
O4^i^—Co1—O5	88.28 (4)	C4—C5—C6	119.71 (12)
O4—Co1—O5	91.72 (4)	C4—C5—H5	120.1
O1^i^—Co1—O5^i^	88.91 (4)	C6—C5—H5	120.1
O1—Co1—O5^i^	91.09 (4)	C6^ii^—C6—C5	120.13 (14)
O4^i^—Co1—O5^i^	91.72 (4)	C6^ii^—C6—C2^ii^	118.74 (14)
O4—Co1—O5^i^	88.28 (4)	C5—C6—C2^ii^	121.14 (11)
O5—Co1—O5^i^	180.00 (8)	O6—C7—N1	124.91 (14)
C1—O1—Co1	130.95 (8)	O6—C7—H7	117.5
C2—O3—H3	107.8 (14)	N1—C7—H7	117.5
Co1—O4—H1	118.7 (16)	N1—C8—H8A	109.5
Co1—O4—H2	117.9 (14)	N1—C8—H8B	109.5
H1—O4—H2	104 (2)	H8A—C8—H8B	109.5
Co1—O5—H6	107.4 (18)	N1—C8—H8C	109.5
Co1—O5—H10	114.6 (14)	H8A—C8—H8C	109.5
H6—O5—H10	105 (2)	H8B—C8—H8C	109.5
C7—N1—C8	122.13 (14)	N1—C9—H9A	109.5
C7—N1—C9	120.50 (14)	N1—C9—H9B	109.5
C8—N1—C9	117.37 (13)	H9A—C9—H9B	109.5
O2—C1—O1	123.35 (11)	N1—C9—H9C	109.5
O2—C1—C3	120.55 (12)	H9A—C9—H9C	109.5
O1—C1—C3	116.08 (11)	H9B—C9—H9C	109.5
O3—C2—C3	123.22 (11)		
			
Co1—O1—C1—O2	12.6 (2)	O1—C1—C3—C4	171.94 (11)
Co1—O1—C1—C3	−168.77 (8)	C2—C3—C4—C5	−1.54 (19)
O3—C2—C3—C4	−177.43 (11)	C1—C3—C4—C5	177.49 (12)
C6^ii^—C2—C3—C4	2.82 (19)	C3—C4—C5—C6	−0.7 (2)
O3—C2—C3—C1	3.5 (2)	C4—C5—C6—C6^ii^	1.6 (2)
C6^ii^—C2—C3—C1	−176.22 (11)	C4—C5—C6—C2^ii^	−178.74 (12)
O2—C1—C3—C2	169.63 (12)	C8—N1—C7—O6	−179.71 (14)
O1—C1—C3—C2	−9.05 (18)	C9—N1—C7—O6	0.7 (2)
O2—C1—C3—C4	−9.39 (19)		

Symmetry codes: (i) −*x*+1, −*y*+1, −*z*+1; (ii) −*x*+2, −*y*+1, −*z*.

### catena-Poly[[[tetraaquacobalt(II)]-µ2-1,5-dihydroxynaphthalene-2,6-dicarboxylato] dimethylformamide disolvate] Hydrogen-bond geometry (Å, º)

**Table:**

*D*—H···*A*	*D*—H	H···*A*	*D*···*A*	*D*—H···*A*
O3—H3···O1	0.78 (2)	1.81 (2)	2.5168 (13)	149.1 (19)
C7—H7···O4^iii^	0.95	2.56	3.2322 (18)	128
O4—H1···O6	0.76 (2)	1.99 (2)	2.7449 (16)	178 (2)
O4—H2···O2^iv^	0.78 (2)	1.96 (2)	2.7284 (15)	168 (2)
O5—H6···O2^i^	0.79 (3)	2.00 (3)	2.7182 (14)	151 (2)
O5—H10···O6^iii^	0.85 (2)	1.95 (2)	2.7836 (15)	166 (2)
C8—H8*C*···C3^i^	0.98	2.85	3.497 (2)	124

Symmetry codes: (i) −*x*+1, −*y*+1, −*z*+1; (iii) −*x*+1, −*y*+2, −*z*+1; (iv) −*x*+2, −*y*+1, −*z*+1.
