# Supplementary figures and images for: Crystal structure of catena-poly[[[tetra­aquacobalt(II)]-μ2-1,5-di­hydroxy­naphthalene-2,6-di­carboxyl­ato] di­methyl­formamide disolvate]
Source: Acta Crystallogr E Crystallogr Commun. 2025 Feb 7;81(Pt 3):204–7. doi: 10.1107/S2056989025000982 (PMC11891581; doi:10.1107/S2056989025000982)

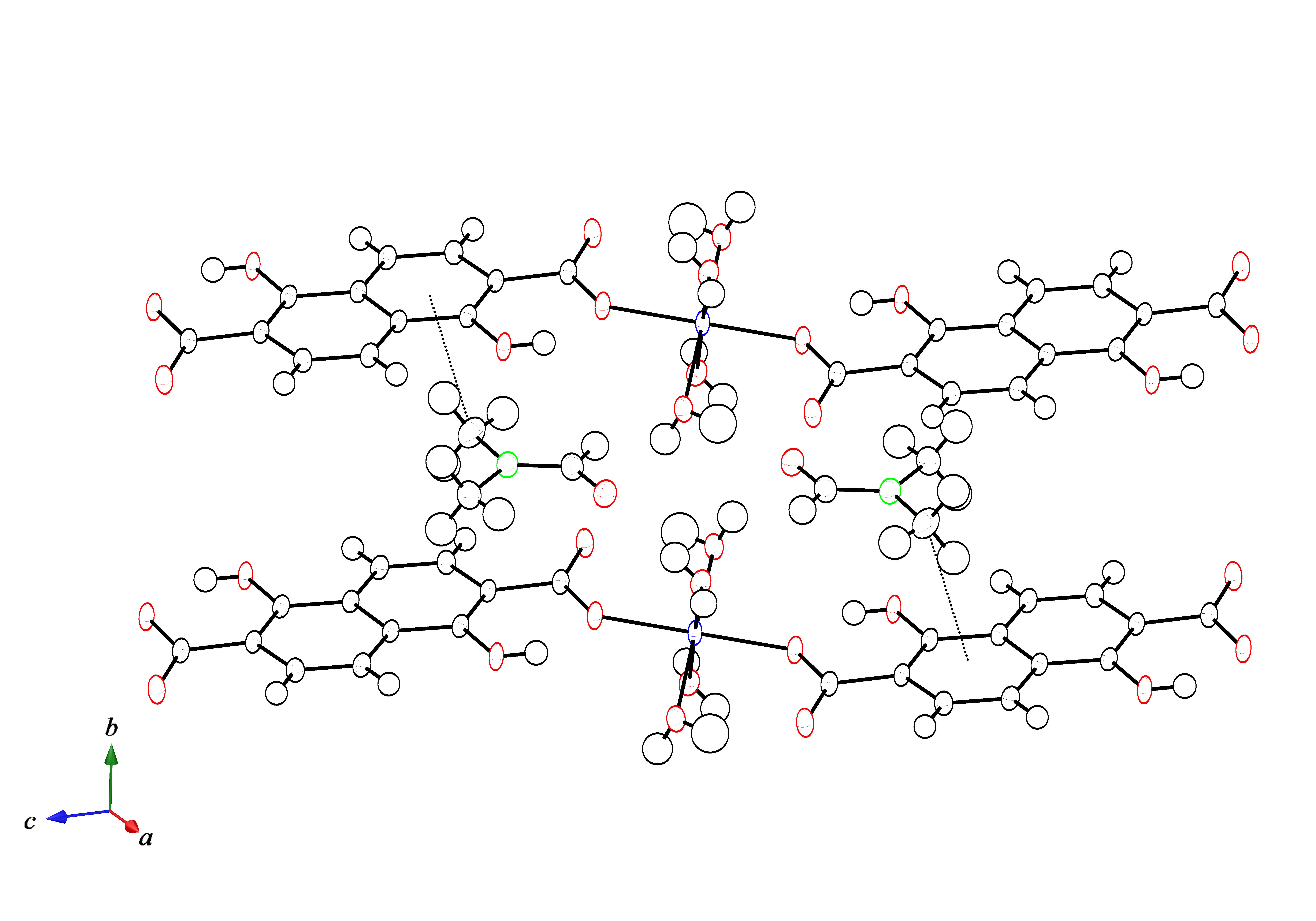

Supplement: Supplementary file 4 [file e-81-00204-sup4.tif]

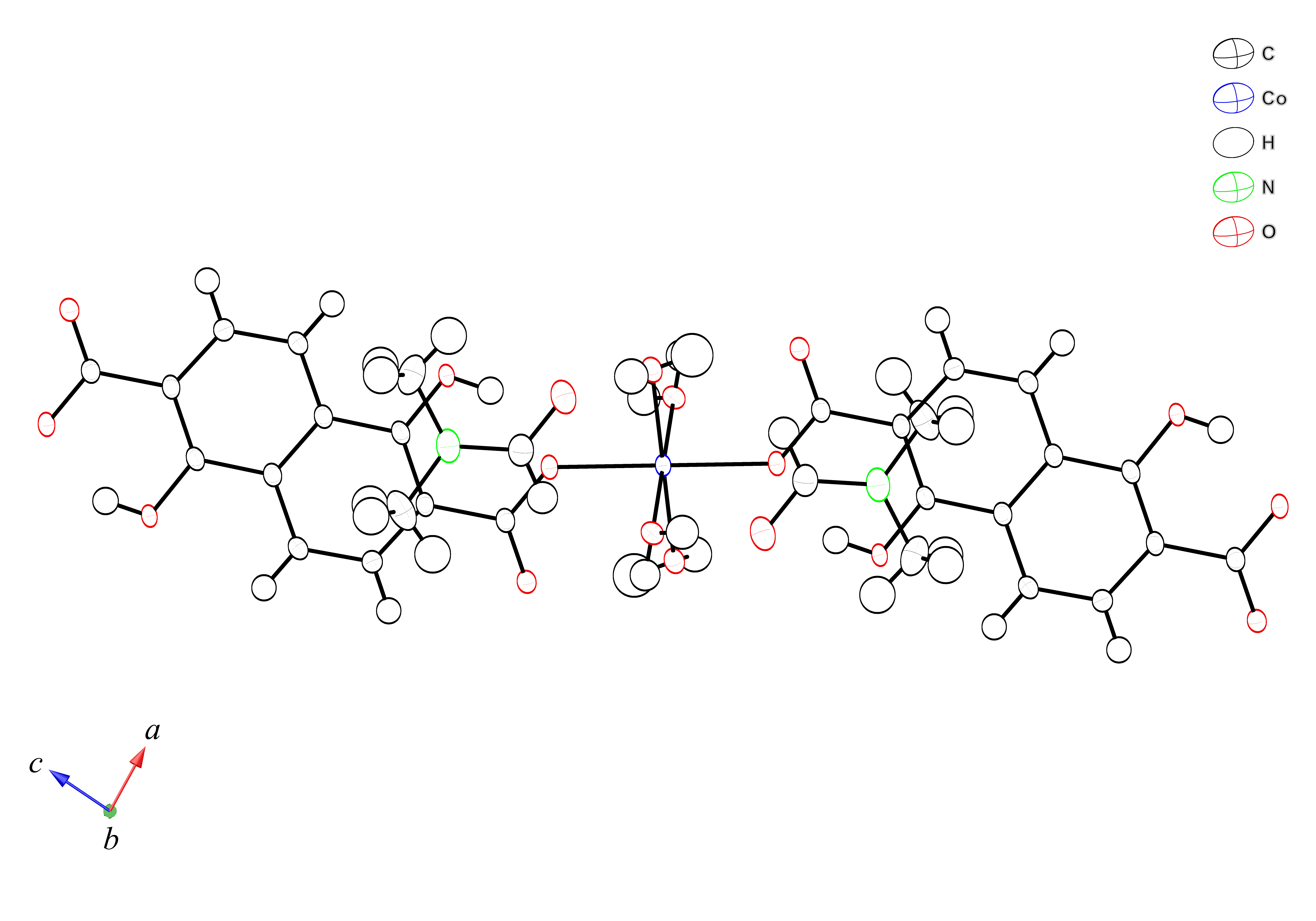

Supplement: Supplementary file 5 [file e-81-00204-sup5.tif]

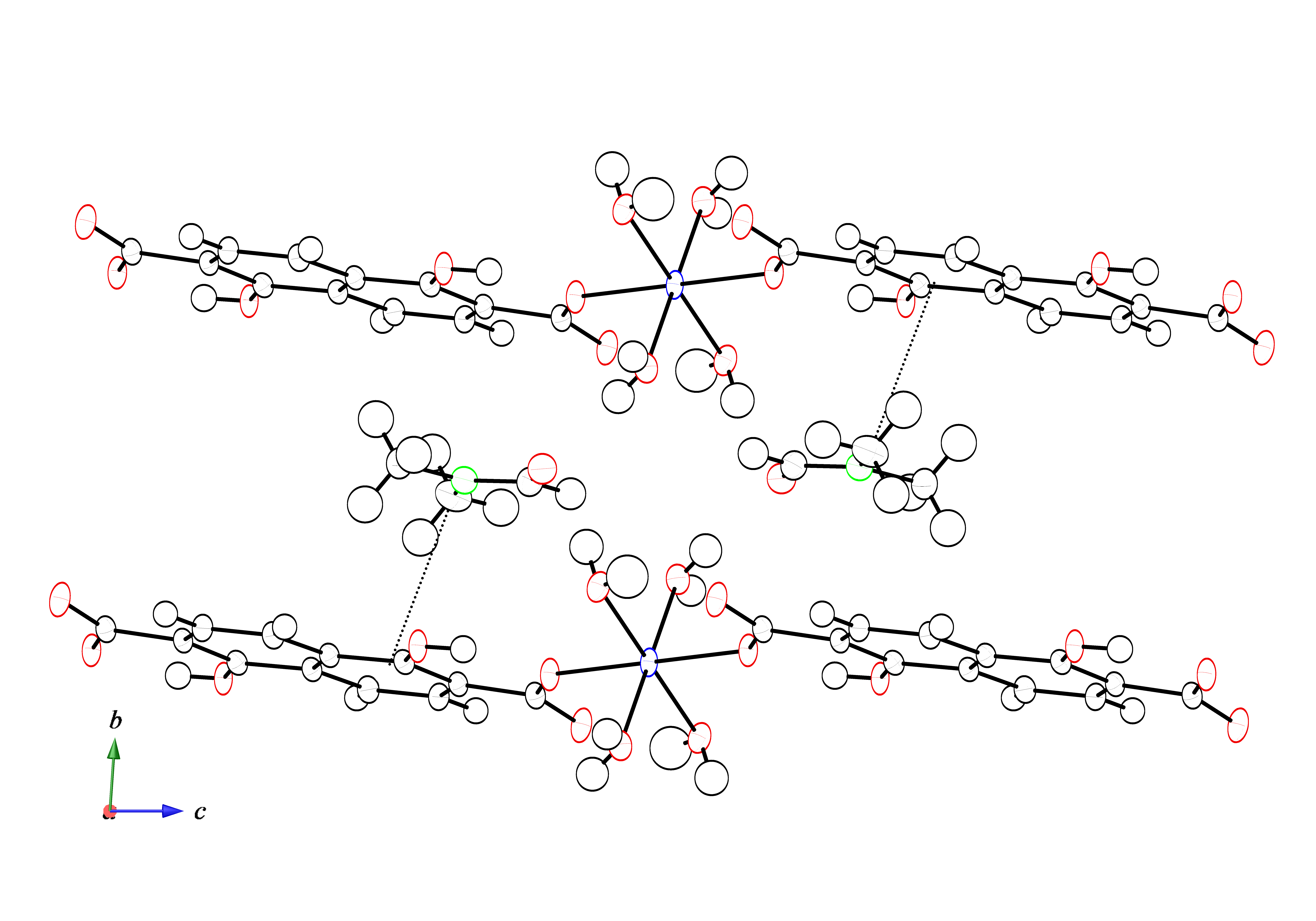

Supplement: Supplementary file 6 [file e-81-00204-sup6.tif]
